# Phenotypically distinct helper NK cells are required for gp96-mediated anti-tumor immunity

**DOI:** 10.1038/srep29889

**Published:** 2016-07-19

**Authors:** Abigail L. Sedlacek, Lauren B. Kinner-Bibeau, Robert J. Binder

**Affiliations:** 1 Department of Immunology, University of Pittsburgh, Pittsburgh, PA 15261 USA

## Abstract

A number of Heat Shock Proteins (HSPs), in the extracellular environment, are immunogenic. Following cross-presentation of HSP-chaperoned peptides by CD91^+^ antigen presenting cells (APCs), T cells are primed with specificity for the derivative antigen-bearing cell. Accordingly, tumor-derived HSPs are in clinical trials for cancer immunotherapy. We investigate the role of NK cells in gp96-mediated anti-tumor immune responses given their propensity to lyse tumor cells. We show that gp96-mediated rejection of tumors requires a unique and necessary helper role in NK cells. This helper role occurs during the effector phase of the anti-tumor immune response and is required for T cell and APC function. Gp96 activates NK cells indirectly via APCs to a phenotype distinct from NK cells activated by other mechanisms such as IL-2. While NK cells have both lytic and cytokine producing properties, we show that gp96 selectively activates cytokine production in NK cells, which is important in the HSP anti-tumor immune response, and leaves their cytotoxic capacity unchanged.

Select members of the heat shock protein (HSP) family are intracellular chaperones of peptides and are immunogenic^1^,^2^. Immune responses elicited by hsp70^3^, calreticulin^4^, hsp90^5^, and gp96^1^,^6^ are specific for the chaperoned (peptide) antigens and have been harnessed for the immunotherapy of cancer^7^,^8^,^9^ and infectious disease^10^. Mechanistically, tumor-derived HSPs in the extracellular environment, as a result of extraneous administration^1^,^3^,^4^,^5^,^6^ or release from necrotizing cells^11^, engage the receptor CD91 on draining lymph node antigen presenting cells (APCs) leading to endocytosis and cross-presentation of the chaperoned peptides to T cells^6^,^12^,^13^. In addition, CD91 initiates signaling cascades within APCs that leads to elaboration of a panel of cytokines and up-regulation of co-stimulatory molecules^11^,^14^. As a singular entity, the HSP-peptide complex leads to priming of T cell responses and tumor rejection. The role of T cell subsets and APCs have been well defined through selective depletions of these cell types in mice^15^.

The study of NK cells in HSP-mediated tumor rejection has been largely correlative and their role in the rejection of tumors remains vague. Immunotherapy of cancer patients with autologous, tumor derived gp96 has been shown to increase the frequency of NK cells in peripheral blood, as well as the expression of their activating receptors and IFNγ following re-stimulation *ex vivo*^16^,^17^. These effects can be enhanced by co-administration of IL-2^18^, an NK cell activating cytokine. In a singular murine study, global deletion of NK cells was sufficient to abrogate rejection of tumors mediated by gp96 in a therapeutic setting^19^. The importance of these observations cannot be underestimated given the clinical application of autologous, tumor-derived HSPs for the immunotherapy of cancer^7^,^8^,^9^.

Although well known for their prominent roles in viral pathogenesis, NK cells in general can be potent mediators in immunotherapy of mouse and human cancers^20^. As versatile lymphoid effectors, NK cells can directly recognize and lyse tumor cells^21^,^22^, but they also control the development and function of adaptive immune responses and tumor immunity^23^,^24^,^25^,^26^,^27^,^28^.

In this study, the prototypical immunogenic HSP, gp96, is used to investigate the role of NK cells in rejection of tumors in murine models of cancer. Using a novel targeted mechanism of temporal depletion of NK cells, we determined that NK cells were essential during the effector phase of tumor rejection, subsequent to gp96 immunization. Although NK cells were necessary for tumor rejection following immunization with gp96, they surprisingly did not show lytic activity towards tumors. Instead, NK cells acted as helper cells and were critical for T cell-mediated tumor lysis. These NK cells were phenotypically distinct from NK cells activated via other mechanisms such as IL-2. This study provides a comprehensive assessment of the role of NK cells in HSP-mediated tumor rejection and reveals a distinct role of these cells that exclusively displays its helper function. These studies suggest that cytotoxic and helper roles, expressed distinctly by CD8^+^ and CD4^+^ T cells respectively in the adaptive immune system, are harbored within the same cell (NK cell) in the innate immune system and are consecutively revealed by differential activation. These studies also have an impact on immunotherapy of cancer.

## Results

### NK cells are required during the effector phase of gp96-mediated tumor rejection

**Figure 1:**
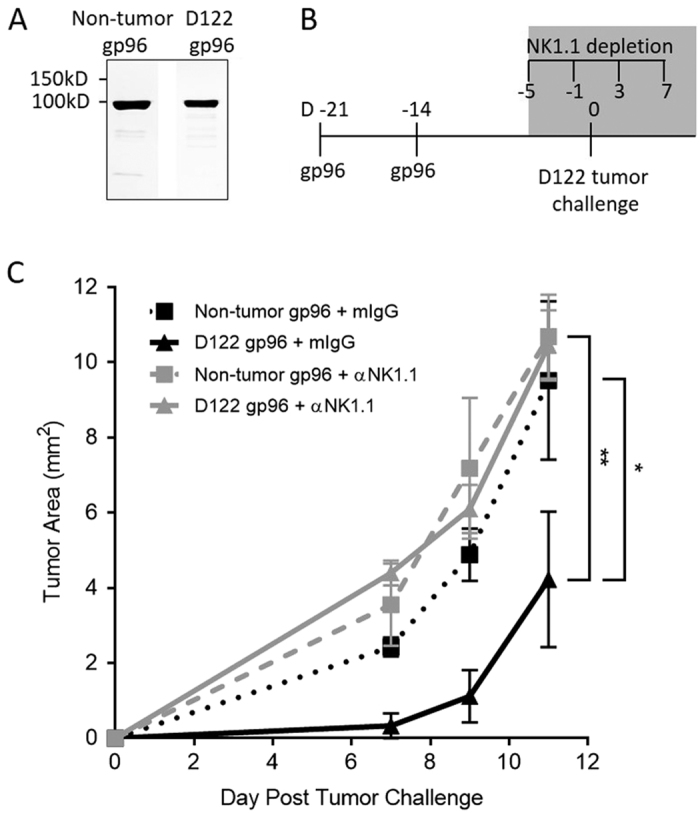
NK cells are necessary during the effector phase of the gp96-mediated anti-tumor immune response. (**A**) Gp96 from tumor cells or normal mouse (non-tumor) tissue was purified to homogeneity as analyzed by SDS-PAGE. (**B**) Mice were immunized twice, one week apart with 2 μg of D122- or non-tumor derived gp96. Prior to tumor challenge with D122 tumor cells and throughout tumor growth mice were treated with anti-NK1.1 or control mIgG as shown. (**C**) Tumor growth was measured over time and is plotted as tumor area. Statistical analysis was performed by ANOVA of area under the curve followed by Bonferroni post-test *p < 0.05, **p < 0.01.

Homogenous tumor-derived gp96 (Fig. 1A) elicits immunity capable of rejecting a subsequent tumor challenge^1^,^6^. In this prophylactic setting, the priming phase (gp96 immunization) is distinct from the effector phase (beginning at tumor challenge) by more than a week, allowing us to specifically target the effector phase. To determine the role of NK cells in such tumor rejection, NK cells were depleted (Fig. S1) in the effector phase of the anti-tumor response, i.e., >1 week following immunization with D122-derived gp96 and 5 days before D122 tumor challenge (Fig. 1B). The delay in tumor development in D122-derived gp96 immunized mice was significantly abrogated when NK cells were depleted compared to mice treated with control IgG (Fig. 1C). Control mice immunized with normal tissue-derived gp96 exhibited no tumor rejection as previously shown. Depletion of NK cells in untreated mice did not affect normal tumor development; growth of tumors was indistinguishable from mice with a full complement of NK cells.

### NK cells do not play a tumor-specific cytolytic role in gp96-mediated tumor rejection

**Figure 2:**
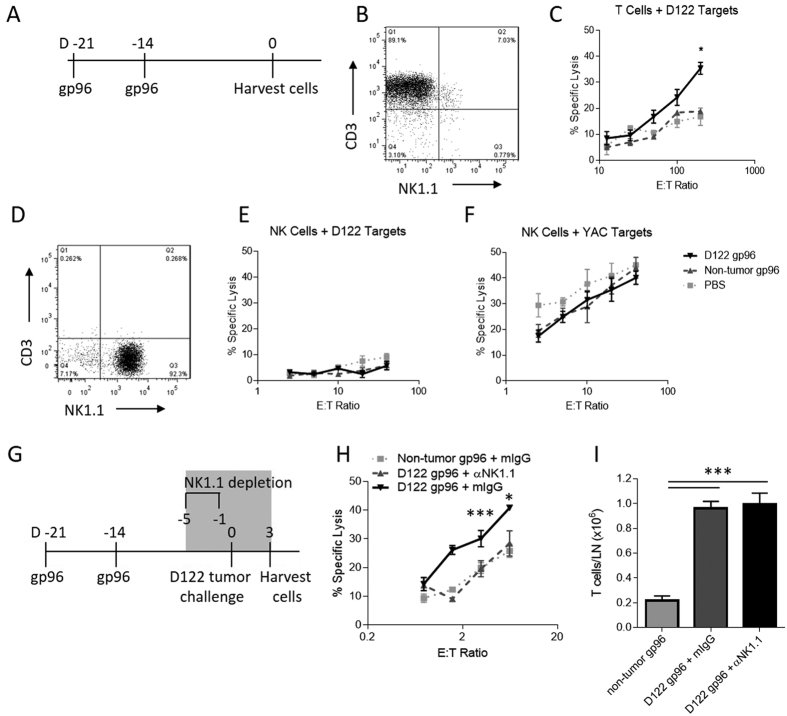
Gp96 activated NK cells do not directly lyse tumor cells but are necessary for tumor- specific CTL function. (**A–F**) Mice were immunized twice, one week apart with 2 μg of D122 or non-tumor derived gp96 and sacrificed 2 weeks later. (**B**) T cells were isolated from the spleens of immunized mice and, (**C**) incubated with labeled D122 target cells in a CTL assay. (**D**) NK cells were isolated from spleens of immunized mice and incubated with (**E**) D122 target cells or (**F**) YAC cells and killing was measured. (**G**) Immunized mice were treated with anti-NK1.1 or mIgG prior to challenge with D122 tumor cells. Three days following challenge with D122, mice were sacrificed and T cells were isolated from draining lymph nodes. (**H**) Cytotoxicity of isolated T cells were assayed using D122 target cells. (**I**) T cells in draining lymph nodes from immunized and challenged mice were counted. Statistical analysis was performed by ANOVA followed by Bonferroni post-test *p < 0.05, **p < 0.01, ***p < 0.001.

NK cells are known for their prominent role in mediating the lysis of target cells, including tumors. Given their role in the effector phase of the immune response (Fig. 1), we hypothesized that NK cells were responsible for tumor cell death, following activation by gp96. Mice were immunized twice weekly with normal tissue- or D122-derived gp96 (Fig. 2A) and two weeks following the last immunization, splenic T and NK cells were isolated (Fig. 2B,D) and analyzed for their cytotoxicity towards D122 cells. T cells isolated from D122-derived gp96 immunized mice were capable of lysing D122 target cells *ex vivo* (Fig. 2C). Control T cells from normal tissue-derived gp96 immunized mice or PBS treated mice were not able to do so (Fig. 2C). In contrast, and surprisingly, NK cells isolated from D122- or non-tumor- derived gp96 immunized mice (Fig. 2D) did not lyse D122 target cells (Fig. 2E) and were comparable to NK cells from PBS treated mice. Importantly, NK cells from all groups retained their lytic capacity though, as they were fully functional in their ability to lyse the NK cell sensitive YAC-1 targets (Fig. 2F). Collectively, these data demonstrate a lack of tumor cytolysis mediated by NK cells following immunization with gp96.

### NK cells display a helper role in gp96-mediated tumor rejection

The requirement for NK cells, and the lack of their cytolytic activity in gp96-mediated tumor rejection, predicted that NK cells were providing a helper role in the effector phase of the immune response, likely by enhancing T cell re-activation and tumor cell killing. To test this prediction, we immunized mice twice weekly with D122-derived gp96 and depleted NK cells before challenging mice with D122 tumor cells to reactivate T cells (Fig. 2G). Mice were sacrificed 3 days following D122 challenge, prior to the formation of palpable tumors. T cells were isolated from draining lymph nodes and used as effector cells to assess T cell killing of D122 target cells *ex vivo*. As shown in Fig. 2H, T cells isolated from mice without NK cell depletion recognized and lysed D122 target cells. In contrast, T cells from mice depleted of NK cells exhibited no increase in lysis of D122 over control, non-tumor gp96 immunized mice. Total T cell numbers from lymph nodes were unaltered by NK cell depletion (Fig. 2I). Thus, while NK cells are required in the effector phase of gp96-mediated tumor rejection, they are not responsible for tumor lysis. Instead, NK cells act to help CTL function.

### Gp96 indirectly activates NK cells via APCs

**Figure 3:**
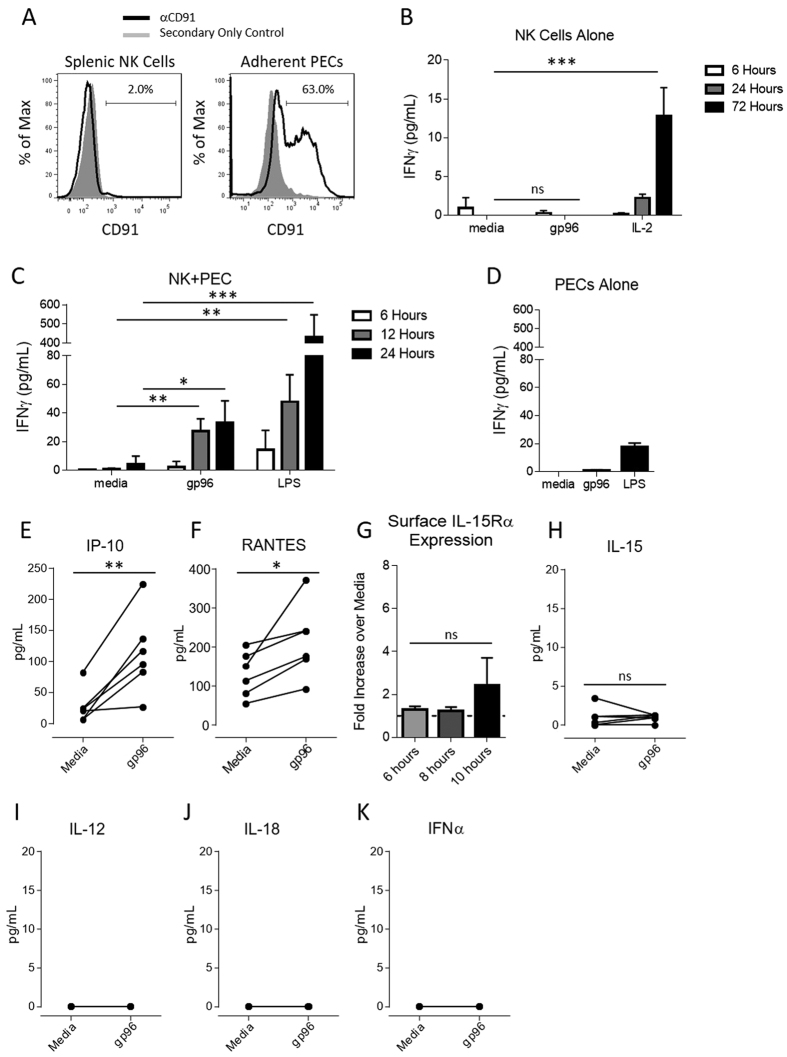
Activation of NK cells by gp96 requires antigen presenting cell intermediaries. (**A**) Naïve splenocytes and adherent PECs were analyzed by flow cytometry. Splenocytes were pre-gated on NK cells (NK1.1^+^, CD3^−^) and CD91 surface expression was determined by flow cytometry. (**B,C**) NK cells were isolated by negative selection and incubated with 200 μg/mL gp96 (**B**) alone or (**C**) with adherent PECs for indicated time points and NK cell activation was measured by ELISA for IFNγ in the supernatant. (**D**) Adherent PECs alone were stimulated with 200 μg/mL gp96 and IFNγ secretion was measured by ELISA. Statistical analysis was performed by ANOVA followed by Bonferroni post-test *p < 0.05, **p < 0.01, ***p < 0.001. PECs were stimulated with 200 μg/mL gp96 for 10 hours unless otherwise indicated. Supernatants were harvested and assayed for IP-10 (**E**), RANTES (**F**), IL-15 (**H**), IL-12 (**I**), IL-18 (**J**) or IFNα (**K**) by Luminex (n = 6) or for surface IL-15Rα expression (**G**) by flow cytometry. Statistical analysis was performed by paired Student’s t-test *p < 0.05, **p < 0.01.

Given the requirement for NK cells in gp96-mediated tumor rejection, we next investigated how NK cells are activated. We have previously identified CD91 as a receptor on APCs for immunogenic HSPs^12^,^13^,^29^. We tested for the expression of CD91 on NK cells by flow cytometry. CD91 was not detected on the surface of murine NK cells (Fig. 3A). We then established a functional assay to determine if gp96 was capable of directly activating NK cells. Freshly isolated, untouched NK cells were incubated with gp96 over the indicated times. NK cell activation was assessed by secretion of IFNγ into the culture supernatant as measured by ELISA. No IFNγ secretion was detected at any time point following gp96 administration (Fig. 3B). As a positive control, IL-2, a known NK cell activator induced robust IFNγ secretion (Fig. 3B). We have previously shown that APCs stimulated with gp96 secrete numerous cytokines and chemokines^13^,^14^. Thus, we investigated whether gp96-activated APCs could indirectly activate NK cells. Adherent peritoneal exudates cells (PECs) which are enriched for macrophage and DC populations and express high levels of CD91 (Fig. 3A), were activated with gp96 and co-cultured with NK cells. At various time points, supernatants were harvested and examined by ELISA for the presence of IFNγ. We were able to detect NK cell activation following exposure to gp96-activated PECs as early as 12 hours post co-culture (Fig. 3C). We did not detect IFNγ in cultures of PECs alone with gp96, confirming NK cells are the source of the secreted IFNγ in the co-cultures (Fig. 3D). Following activation by gp96, PECs secrete IP-10 (Fig. 3E) and RANTES (Fig. 3F), molecules previously reported to activate NK cells. However, gp96-stimulated PECs did not increase expression of surface IL-15Rα (Fig. 3G), nor release IL-15 (Fig. 3H), IL-12 (Fig. 3I), IL-18 (Fig. 3J) or IFNα (Fig. 3K), which are other classical cytokines reported to be potent activators of NK cells. BMDCs, which mimic cytokine release by adherent PECs following gp96 stimulation^13^,^14^, similarly activate NK cells (data not shown). These experiments demonstrate that NK cells are activated by gp96 indirectly via antigen presenting cells.

### NK cells activated by gp96 are phenotypically distinct from lymphokine activated killer cells

**Figure 4:**
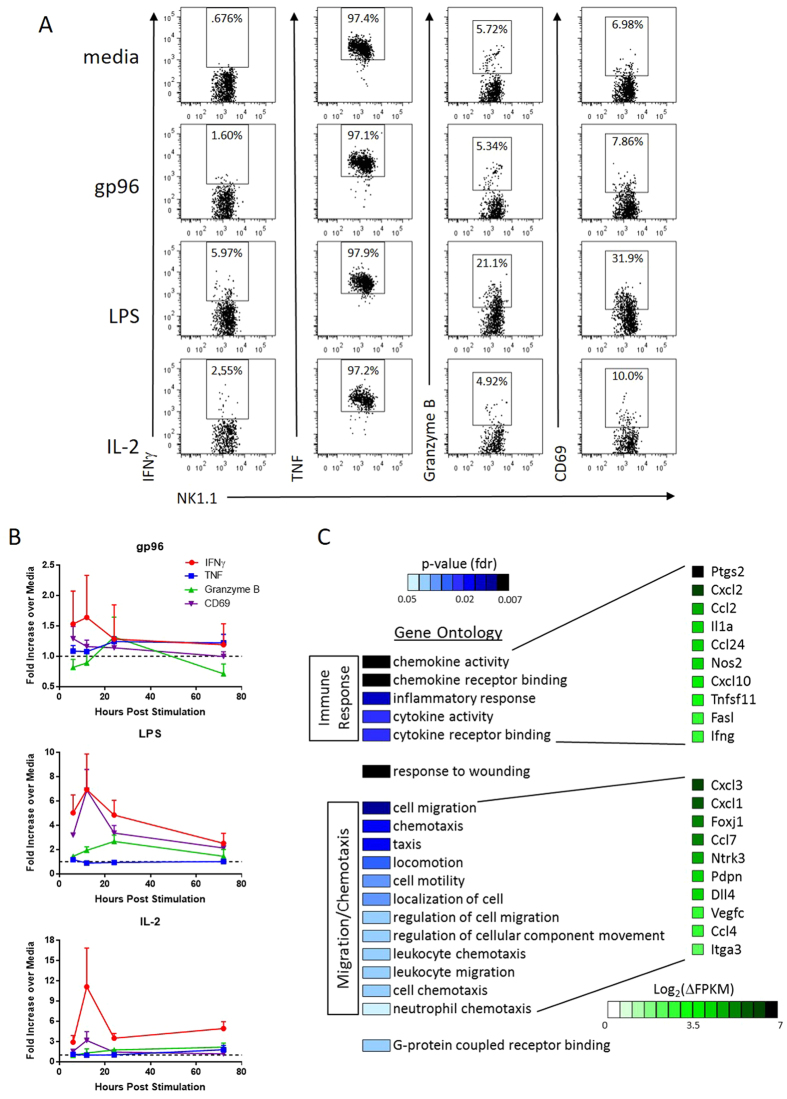
Indirect activation by gp96 results in an NK cell profile distinct from lymphokine activated cells. NK cells were cultured with PECs in the presence of gp96, LPS or IL-2. Markers of NK cell effector function and activation were measured over time. (**A**) Flow cytometry analysis of NK cells in culture 24 hours post stimulation. Graphs were pre-gated on NK1.1^+^ cells, and intracellular expression of IFNγ, TNF-α, Granzyme B, and surface expression of CD69 were analyzed. (**B**) Expression of effector/activation markers was analyzed by flow cytometry as above relative to media control samples and plotted over time for NK cells stimulated with gp96, LPS or IL-2. n = 3 mice from one representative experiment of 2 experiments. (**C**) NK cells were re-isolated from 12 hour co-cultures and RNA was harvested for RNA-sequencing analysis. The top 500 genes increased by gp96 activation were analyzed by gene ontology analysis and the ontologies that were significantly represented are indicated along with level of significance and representative genes.

NK cells are routinely activated by IL-2 in the presence of APCs to obtain lymphokine activated killer cells^30^, characterized by their expression of IFNγ and CD69, and cytolytic function. A similar phenotype can be achieved with LPS stimulation via TLR4 on APCs^31^. We compared NK cells activated (in the presence of APCs) with IL-2 or LPS to gp96 with respect to expression of various activation markers. NK cells activated by gp96 were IFNγ^+^ and TNF-α^+^ as determined by intracellular staining but did not express CD69 or increased granzyme B (Fig. 4A). This was distinct from NK cells activated by IL-2 or LPS (IFNγ^+^, TNF-α^+^, CD69^+^, granzyme B^low/+^). These markers were monitored over time up to 72 hrs (Fig. 4B). In addition to examining markers of activation, IL-2 and LPS- activated NK cells are functionally tumor cell lytic^20^,^21^,^22^, while in contrast, NK cells activated by gp96 do not increase tumor cell lysis (Fig. 2E).

In addition to flow cytometric analysis, we re-isolated NK cells from NK-APC co-cultures in the presence or absence of gp96 and harvested RNA for transcriptome analysis by RNA-seq. NK cells were activated with gp96 for 12 hours, the earliest time point we observed IFNγ secretion (Fig. 3C). Gene ontology analysis was performed on the top 500 genes with increased or decreased expression following gp96 activation. Genes that were activated following gp96 activation are primarily enriched (false discovery rate <0.05) for those known to induce immune responses and enhance chemoattraction (Fig. 4C). Interestingly, several of the chemokines identified in the gene ontology analysis are those that are involved in the recruitment of T cells (Cxcl10, Ccl24) as well as APCs (Ccl2, Ccl24), further implicating an NK cell helper phenotype. The genes that were decreased following gp96 activation did not map to a particular gene ontology.

### Gp96 mediated cross-talk between NK cells and APCs augments APC cross-presentation ability

**Figure 5:**
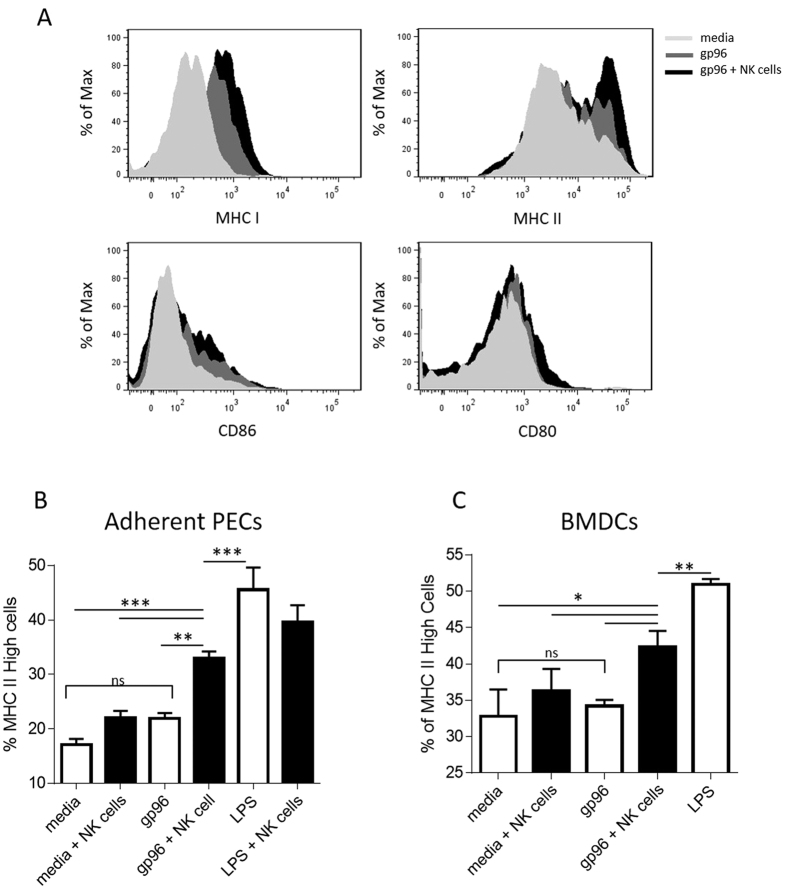
NK cells activated indirectly by gp96 enhance antigen presenting cell cross-priming ability. PECs or BMDCs were stimulated with gp96 in the presence or absence of NK cells and markers of cross-priming were assessed on CD11b^+^ cells by flow cytometry. (**A**) PECs were stimulated with optimal doses (200 μg/mL) of gp96. MHC I, MHC II, CD86, and CD80 expression was analyzed by flow cytometry. PECs (**B**) or BMDCs (**C**) were stimulated with suboptimal doses (100 μg/mL) of gp96. MHC II expression was measured as a readout for APC activation. Statistical analysis was performed by ANOVA followed by Bonferroni post-test *p < 0.05, **p < 0.01, ***p < 0.001.

The pleiotropism associated with IFNγ (released in this case by NK cells) as well as the gene ontology analysis, led us to investigate feedback effects on APCs in co-cultures after 48 hours. PECs co-cultured with NK cells and gp96 expressed increased levels of MHC I, MHC II and CD86 but not CD80 compared to APCs incubated with gp96 or media alone (Fig. 5A). When suboptimal amounts of gp96 are used to stimulate PECs only MHC II is upregulated and only in the presence of NK cells (Fig. 5B) indicating a titration in the effect of gp96 on APCs and the feedback role of NK cells. These effects occur across multiple APC types including BMDCs (Fig. 5C).

## Discussion

We have investigated the role of NK cells in gp96-mediated anti-tumor immunity and discovered that gp96 is able to prime a unique and necessary helper role in NK cells for CTL-function during the effector arm of the immune response. Gp96 does not directly activate NK cells, rather NK cell activation occurs indirectly via APCs expressing the HSP receptor, CD91. In other reported systems, NK cell activation by APCs is classically driven by IL-12, IL-15, and IL-18^20^. None of these cytokines were detected from PECs stimulated with 200 μg/ml of gp96. However, other NK cell activators exist. At high doses, IP-10 activates naïve NK cells to become lytic and improve T cell proliferation^32^. Indeed, gp96-stimulated APCs released low-moderate levels of IP-10 and the NK cells thus activated only had a helper phenotype. The program initiated in NK cells that decides the lytic or helper fate of activated NK cells could therefore be (partially) dependent on the dose of IP-10 released by the APC. In addition to cytokine activation, NK cells are also activated by receptor-ligand interactions and the contribution of these receptor-ligand interactions between the NK cells and APCs should be appreciated as well^33^,^34^,^35^.

NK-APC-T cell interactions are dynamic and mutually beneficial, leading to NK and T cell activation and enhancement of APC cross-presentation ability. We observe this phenomenon in NK cell – APC co-cultures which is mediated by gp96. In agreement with this concept, NK cells activated by gp96 were observed to upregulate the expression of many cytokines and chemokines associated with the enhancement of APCs and T cells. In addition to these soluble factors, we cannot exclude a role for cell-cell interactions which may also regulate APC and T cell activation. While gp96 alone activates APCs and enhances their ability to cross-prime antigens, the markers of such activation are significantly enhanced in the presence of NK cells. Two observations deserve highlighting; (i) the ability of NK cells to improve APC maturation does not extend to all maturation markers. Of those that were investigated, only the maturation markers that are induced by gp96 itself via CD91 were upregulated (i.e., only improves a basal program of APC activation initiated by gp96) and (ii) NK cell feedback activation does not globally increase all facets of APC maturation. This queries the emphasis placed on the pleiotropism of IFNγ^20^,^36^, released by activated NK cells, in such feedback mechanisms. Since not all IFNγ-sensitive genes, e.g., CD80, are altered in co-cultures with gp96, there must be a customized regulatory mechanism dictating the cross-priming program. These mechanisms may include receptor-ligand interactions or additional unidentified cytokines and are currently under investigation.

NK cell memory is qualitatively similar to that in B and T lymphocytes^37^. We have not ruled out the activation of NK cells during the priming phase of the gp96-mediated immune response, despite their required function during the tumor rejection phase. Our studies also highlight the interesting concept that cytotoxic and helper roles are harbored within NK cell that can be manifested discretely by differential activation programs, and may indeed be present in memory NK cells.

Recent Phase III clinical trials using autologous, tumor-derived gp96 on patients with renal cell carcinoma or melanoma did not achieve the overall stated endpoints although a subset of patients, those with early disease stage, did benefit from the vaccine to survive longer than those in the control arm^8^,^9^. While a role for T cells in HSP cancer immunotherapy has been confirmed^38^,^39^ a role of NK cells has not been considered in any of these trials. With our current report, we envisage therapeutically enhancing gp96-mediated immune responses by co-administration of NK cell augmenting regimens such as IL-2 therapy for enhanced clinical benefit.

## Materials and Methods

### Mice and Cell lines

Female C57BL/6 mice were purchased from The Jackson Laboratory (Bar Harbor, ME) under experimental protocols approved by the University of Pittsburgh Institutional Animal Care and Use Committee (IACUC) and experiments were performed in compliance with these IACUC guidelines. D122, a clone of the Lewis Lung Carcinoma, was cultured in complete DMEM containing 1% sodium pyruvate, 1% L-glutamine, 1% nonessential amino acids, 1% penicillin and streptomycin, 0.1% 2-mercaptoethanol, and 10% FBS (GIBCO).

### Gp96 purification

Gp96 was isolated from naïve murine livers (non-tumor gp96) or cultured D122 cells (D122-derived gp96) as previously described^1^.

### Immunization and tumor challenge

Mice were immunized twice, one week apart each with 2 μg gp96 injected intradermally on the ventral side. Two weeks following the second gp96 immunization, mice were challenged with 5 × 10^4^ D122 tumor cells intradermally dorsally. Tumor growth was monitored 2–3 times weekly by caliper measurements and are plotted as tumor area: 3.14 × (mean diameter/2)^2^.

### NK cell depletion

The PK136 hybridoma was a kind gift from Dr. Edith Lord (University of Rochester, NY). Antibody was purified from hybridoma supernatants by Melon Gel chromatography. Mice received 200 μg/mouse of αNK1.1 or control mIgG intraperitoneally every 4 days starting 5 days prior to tumor challenge. NK cell depletion was complete within 24 hrs, and remained undetected throughout tumor growth (Fig. S1).

### Cell isolation

All *ex vivo* isolated cells were cultured in RPMI containing 1% sodium pyruvate, 1% L-glutamine, 1% nonessential amino acids, 1% penicillin and streptomycin, 0.1% 2-mercaptoethanol, and 5% FBS (GIBCO). Both NK and T cells were isolated using MACS kits with untouched protocols; NK cells were isolated by negative selection using the NK cell isolation kit II and T cells were isolated by negative selection using the Pan T cell isolation kit II. Isolations typically yielded >80% purity. Total peritoneal exudate cells (PECs) were isolated by peritoneal lavage of *naïve* mice with sterile PBS and were plated overnight for adherence. No inflammatory agent was administered. The next day, non-adherent cells were removed to yield adherent PECs. Bone marrow-derived dendritic cells (BMDCs) were generated by culturing 2–4 × 10^6^ bone marrow cells in the presence of 20 ng/mL GMCSF for 6–7 days, supplementing cultured cells with fresh RPMI and GMCSF on day 3.

### Flow Cytometric Analysis

Spleen and lymph nodes were processed into single cell suspensions by physical dissociation and treated with Fc Block (BD Biosciences). Samples were stained for 20 minutes in PBS with 1% BSA and 0.1% sodium azide. Intracellular staining was performed with Cytofix/Cytoperm (BD Biosciences) prior to intracellular staining for 40 minutes. Samples were analyzed using a LSR II or Fortessa cytometer (BD Biosciences) and FlowJo software version 7.6 (Tree Star Inc.). The antibodies used were anti-CD3ε (clone 145-2C11), anti-CD11b (clone M1/70), anti-CD69 (clone A1-2F3), anti-CD80 (clone 16-10A1), anti-CD86 (GL1), anti-CD91 (clone 5A6), anti-granzyme B (clone NGZB), anti-H2K^b^ (clone AF6-88.5), anti-I-A/I-E (clone M5/114.15.2), anti-IFNγ (clone XMG1.2), anti-NK1.1 (clone PK136), and anti-tumor necrosis factor α (TNF-α) (clone MP6-XT22).

### Immunological assays

IFNγ ELISAs were performed with Mouse IFNγ Femto-HS Ready-Set-Go kit (eBioscience). Cell-Mediated Cytotoxicity Assays were performed using Live/Dead Cell-Mediated Cytotoxicity Kit (Molecular Probes) following 4 hour incubations. Specific lysis was determined by; 100 x (Experimental Lysis–Background Lysis/100–Background Lysis). Luminex (eBioscience) consisted of the single analyates: IL-12, IL-18, IL-15/IL-15R, IFNα, IP-10, and RANTES. Samples were analyzed using a Bioplex II Luminex machine.

### RNA-seq

Samples were prepared using Clontech RiboGone (634846) and SMARTer Universal Low Input RNA kit (634938) for rRNA depletion and cDNA synthesis. cDNA libraries were prepared using the IonTorrent Ion Plus fragment library kit (4471252) and Ion Xpress barcode adaptor kit (4471250). Templates were prepared using Ion PI Hi-Q Chef kit (A27198) on the Ion Chef platform and sequenced using the Ion Proton on an Ion PI v3 chip.

### Mapping and analysis of RNA-seq

Adaptor sequences and low quality reads were trimmed using Fastq Trimmer^40^. Reads were mapped to the murine reference genome (mm9) using TopHat software package^41^ and annotation of known Ensembl transcripts was obtained from Illumina igenomes on the Galaxy database. FPKM values were calculated with Cufflinks and differentially expressed genes were analyzed using Cuffdiff^42^. Genetrail^43^ was used for gene ontology analysis to test for enrichment of gene families associated with differentially expressed genes. We performed this analysis using default parameters set by Genetrail software and show enrichment results requiring 5+ genes per family.

### Statistical analysis

Statistical analysis was run using GraphPad Prism software version 6. Student’s t-test, area under the curve analysis, and analysis of variance (ANOVA) followed by the indicated post-test was used where indicated. P < 0.05 was considered significant.

## Additional Information

**How to cite this article**: Sedlacek, A. L. *et al*. Phenotypically distinct helper NK cells are required for gp96-mediated anti-tumor immunity. *Sci. Rep.* **6**, 29889; doi: 10.1038/srep29889 (2016).

## Supplementary Material

Supplementary Information
