# Supplementary material for: Phenotypically distinct helper NK cells are required for gp96-mediated anti-tumor immunity
Source: Sci Rep. 2016 Jul 19;6:29889. doi: 10.1038/srep29889 (PMC4949418; doi:10.1038/srep29889)
Supplement: Supplementary Information [file srep29889-s1.pdf]

Supplemental Information

Phenotypical distinct helper NK cells are required for gp96-mediated anti-tumor immunity

Sedlacek AL, Kinner-Bibeau LB, and Binder RJ

A

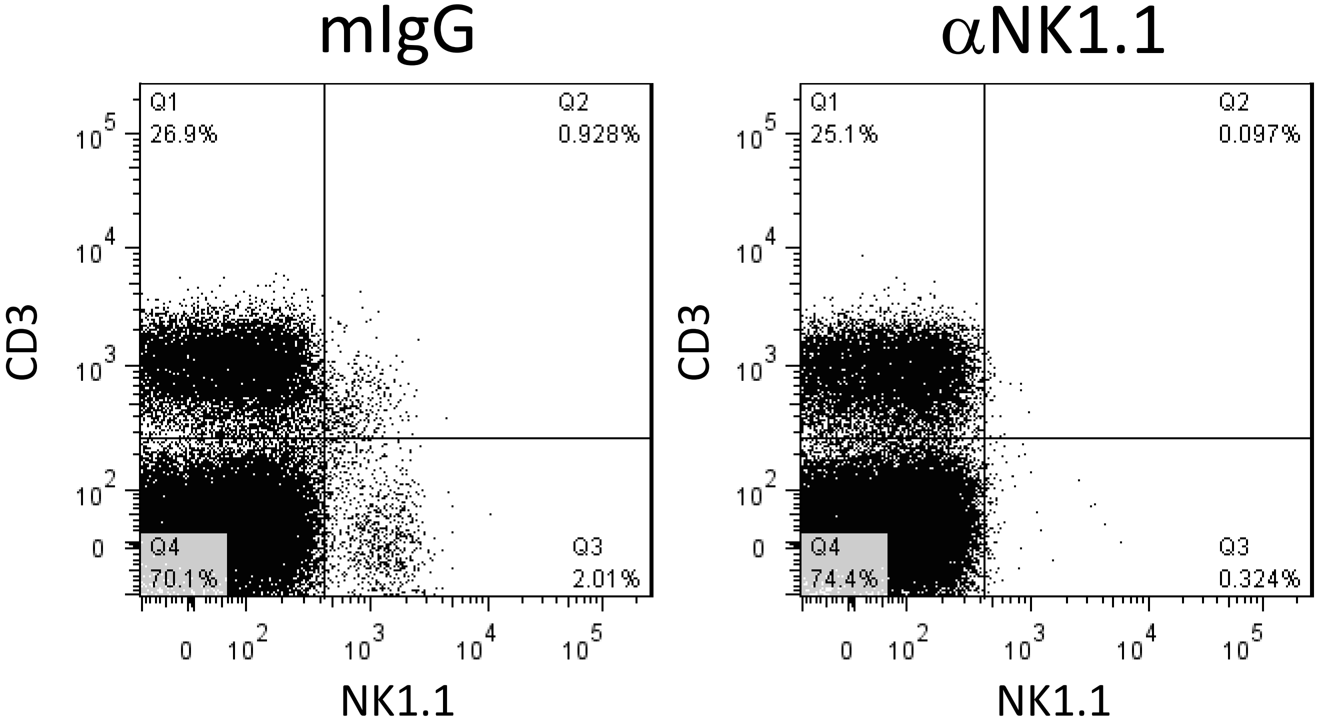
mIgG
αNK1.1
Q1
26.9%
Q2
0.928%
Q3
2.01%
Q4
70.1%
Q1
25.1%
Q2
0.097%
Q3
0.324%
Q4
74.4%
CD3
NK1.1
0
10^2
10^3
10^4
10^5

B

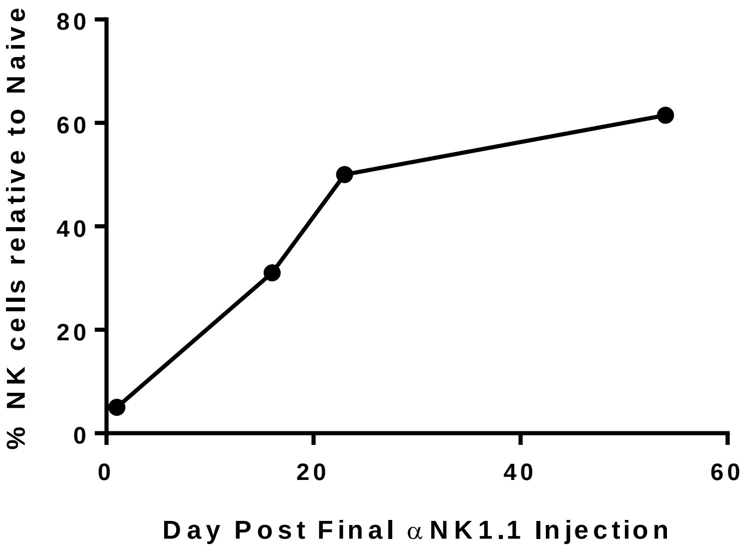
% NK cells relative to Naive
Day Post Final αNK1.1 Injection
0
20
40
60
80

Supplemental Figure 1. **Whole mouse depletion of NK cell cells is specific, durable and long lasting.** Mice treated with anti-NK1.1 were assessed for NK cell depletion by flow cytometric analysis of spleens. (A) Mice were treated with anti-NK1.1 as in Fig 1B and sacrificed on Day 0 to determine NK cell frequency. (B) NK cell recovery over time was assessed by monitoring splenic NK cell frequencies relative to naïve mice.
